# 1254. DoD Adherence to CDC Core Elements of Hospital Antibiotic Stewardship Programs

**DOI:** 10.1093/ofid/ofad500.1094

**Published:** 2023-11-27

**Authors:** LeeAnne C Lynch, David R Tribble, David R Tribble, Katrin Mende

**Affiliations:** Uniformed Services University of the Health Sciences, Silver Spring, Maryland; Uniformed Services University of the Health Sciences, Silver Spring, Maryland; Uniformed Services University of the Health Sciences, Silver Spring, Maryland; Brooke Army Medical Center, San Antonio, Texas

## Abstract

**Background:**

In response to the 2014 Executive Order on Combating Antibiotic-Resistant Bacteria, the Centers for Disease Control and Prevention (CDC) released “Core Elements of Hospital Antibiotic Stewardship Programs” in 2014 and updated it in 2019. In 2017, the Department of Defense (DoD) required hospitals to establish antimicrobial stewardship programs (ASPs) that include CDC Core Elements (CEs). DoD ASPs are often evaluated at the facility level but have not yet been assessed system-wide. This study aims to analyze DoD ASPs as an enterprise to inform future quality improvement.

**Methods:**

Data from the CDC’s National Healthcare Safety Network (NHSN) hospital annual survey, which includes self-reported responses about facility characteristics and various hospital programs (including ASPs), were analyzed in Stata/IC 16.1. A framework analysis was conducted to determine how ASPs are structured, using CDC CEs as a guide. DoD adherence for each individual and all CEs was compared to national data for 2017-2021 and averaged over the same time frame. Adherence was measured using CDC methodology: a positive response to > 1 question within a CE was categorized as that CE being met.

**Results:**

From 2017-2021, between 4,940 and 5,053 hospitals responded to the NHSN annual survey, including 46 to 48 DoD hospitals (1.0% of reporting facilities). Reporting facility participation varies year-to-year, but average percent CE adherence nationally and for the DoD showed education, reporting, and tracking had the lowest adherence (Table 1; Figure 1). DoD differed from national adherence for the CEs of education (-1.9%) and action and tracking (-1.3%). All CEs were met for 82.7% of DoD hospitals compared to 87.2% of hospitals nationally.

**Figure:**
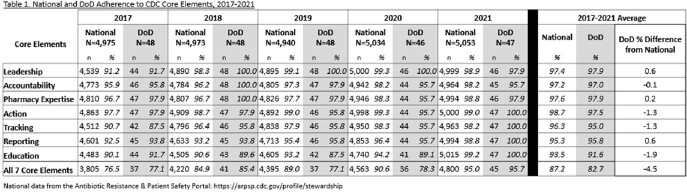

**Figure:**
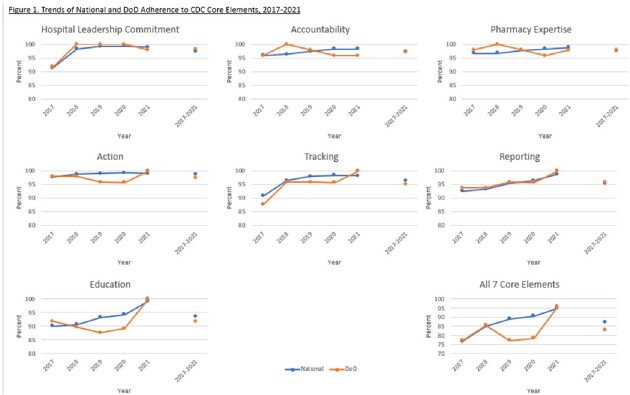

**Conclusion:**

National and DoD CEs adherence levels both showed the largest 2017-2021 average deficiencies in education, followed by tracking and reporting. Differences between national and DoD adherence levels were observed for several CEs. The DoD sample size is small and adherence was variable. However, these findings suggest that the CEs where DoD lags national adherence - education, action, and tracking - present opportunities for the DoD to improve its ASPs. A similar comparison with national adherence percentages may also prove useful for other health systems to undertake.

**Disclosures:**

**All Authors**: No reported disclosures

